# Mechanical Performance of Plywood TIE Joints Under Tension and Shear in the WikiHouse Skylark Modular System

**DOI:** 10.3390/ma18204738

**Published:** 2025-10-16

**Authors:** Moisés Sandoval, Juan Pablo Cárdenas-Ramírez, Paula Soto-Zúñiga, Michael Arnett, Angelo Oñate, Jorge Leiva, Rodrigo Cancino, Víctor Tuninetti

**Affiliations:** 1 Master Program in Engineering Sciences, Faculty of Engineering, Universidad de La Frontera, Temuco 4811230, Chile; m.sandoval27@ufromail.cl; 2 R&D Innovation Department, Eagon Lautaro S.A., Ruta 5 Sur Km 644, Lautaro 4860000, Chile; rcancino@eagon.cl; 3 Escuela de Construcción Civil, Facultad de Ingeniería, Universidad de Valparaíso, General Cruz 222, Valparaíso 2341266, Chile; juanpablo.cardenas@uautonoma.cl; 4 Program of Civil Engineering, Universidad de La Frontera, Temuco 4811230, Chile; p.soto10@ufromail.cl; 5 Facultad de Arquitectura, Construcción y Medio Ambiente, Universidad Autónoma de Chile, Temuco 4810101, Chile; michael.arnett@uautonoma.cl; 6 Department of Materials Engineering (DIMAT), Faculty of Engineering, Universidad de Concepción, Edmundo Larenas 315, Concepción 4070138, Chile; aonates@udec.cl; 7 Doctoral Program in Engineering, Universidad de La Frontera, Temuco 4811230, Chile; 8 Department of Mechanical Engineering, Universidad de La Frontera, Temuco 4811230, Chile

**Keywords:** modular timber construction, WikiHouse skylark, plywood TIE joints, sustainable housing systems, fire-resistant plywood, weather-resistant plywood, tensile and shear performance, strength–ductility trade-off, prefabricated structural systems, open-source construction design

## Abstract

The construction sector’s environmental footprint is driving the adoption of sustainable modular timber systems. The WikiHouse Skylark is a promising open-source model whose structural reliability depends on the performance of its critical plywood TIE joints. This study presents an experimental investigation of full-scale TIE joints fabricated from 18 mm Pinus radiata plywood in three variants: Standard (STD), Weather-Resistant (HR), and Fire-Resistant (FR). Monotonic tensile and shear tests were conducted to evaluate load–displacement behavior and failure modes. While the mean ultimate strengths varied between panel types, with HR highest in tension (7.7 kN) and FR highest in shear (8.2 kN), the most critical finding was the effect of the treatments on failure mode. The FR treatment induced a brittle fracture with significantly reduced ductility, in contrast to the more ductile tearing observed in STD and HR panels. This highlights a clear strength–ductility trade-off introduced by the fire-retardant treatment, a key consideration for structural design in modular timber construction. This dataset provides an essential empirical foundation for the numerical modeling and design guidelines of WikiHouse TIE joints, advancing the development of resilient and sustainable prefabricated housing.

## 1. Introduction

The building and construction sector plays a fundamental role in the global economy. However, due to its scale and low efficiency, it was estimated that, in 2023, this sector accounted for 34% of total global energy consumption and emissions [1,2,3]. The slow integration of Industry 4.0 technologies impedes the adoption of systematic methodologies and hinders efforts to mitigate environmental impacts and promote circularity [4,5]. For instance, in the case of concrete buildings, cement production alone is estimated to contribute between 5% and 8% of global annual CO_2_ emissions [6,7,8,9].

Currently, the integration of digital tools and technologies (DTT) such as Life Cycle Assessment (LCA) and Building Information Modeling (BIM) helps reduce uncertainty regarding environmental impact and economic viability at early design stages [5,10,11]. This represents an opportunity to iteratively improve both environmental and economic performance based on the analysis of monitored variables and reliable quantitative results [12,13]. In this context, prefabrication and modular construction (MC) methodologies are gaining momentum by enabling the production of higher-quality and more efficient buildings. These approaches systematically integrate principles such as Design for Disassembly (DfD) and Design for Manufacturing and Assembly (DfMA), allowing for reductions in emissions, waste, and on-site execution times [14,15,16]. For example, Tavares et al. [17] used LCA to demonstrate that implementing prefabricated systems in steel, concrete, or wood for single-family homes reduces environmental impacts in all cases. Similarly, Hu et al. [18] analyzed 412 construction projects in China, 61 of which used prefabrication, and concluded that current technology can reduce waste generation by 25.85% compared to traditional construction methods.

Another promising approach is the use of wood, especially when combined with the aforementioned technologies. Kumar et al. [19] showed that applying the 2021 International Building Code (IBC) regulations can reduce the Global Warming Potential (GWP), by 39–51% compared to reinforced concrete and by 28–34% compared to steel, when using mass timber (MT) solutions. Like any process of research, development, and innovation (RDI), adjustments and improvements must be informed by empirical feedback. One of the main challenges facing these methodologies is ensuring robust structural performance that meets both design and transportation demands, while aligning with the competencies and capabilities of the companies responsible for execution [20,21,22]. However, many modular construction systems still lack sufficient understanding of their structural performance and, as a result, lack specific design guidelines [23,24]. This issue becomes more critical in complex projects such as high-rise buildings or structures subjected to significant lateral loads (e.g., wind or earthquakes) [25,26]. The challenge is particularly acute in seismic countries such as Chile, where building codes have raised minimum structural design requirements based on empirical evidence from large-magnitude earthquakes, such as the 1960 Valdivia earthquake (Mw 9.5) and the 2010 Maule earthquake (Mw 8.8) [27,28].

The urgent need to develop intelligent, climate-resilient solutions creates an opportunity to foster research in prefabricated and modular timber construction systems. Globally, these systems have been widely used for centuries, and timber joints in particular have historically demonstrated reliable structural behavior under both static and dynamic loads [29,30,31,32]. Pan et al. [33] analyzed the dynamic behavior and seismic response of a historic timber structure on a slope in China through quasi-static cyclic tests of a full-scale mortise–tenon joint and finite element simulations. To balance computational demand with structural accuracy, non-critical elements were modeled as continuous elastic beams, while joints were discretized in detail. Kłosowski et al. [34] studied dovetail and saddle-notch joints, highlighting the importance of boundary conditions and material properties in their stochastic numerical analysis. While the diversity of timber joints is too broad for complete characterization, understanding typical connection behavior through stress distribution and failure mode analyses provides a valuable foundation [35]. Rad et al. [36] examined a CNC-machined through-tenon joint made of Laminated Veneer Lumber (LVL) in tensile tests, concluding that insertion angle (45° vs. 60°) and fiber orientation strongly influenced maximum strength (Fmax) and failure mode. Their results showed higher resistance in parallel specimens (Fmax = 9.0 kN and 14.65 kN for 45° and 60°, respectively) compared to perpendicular specimens (~6 kN). They also observed that 60° joints exhibited superior performance by combining tensile and shear failure modes, unlike 45° joints that showed only shear failure. This focus on component-level analysis to understand structural behavior is a common theme, with other studies investigating the static performance of strengthened timber beams [37]. These works collectively show that component geometry, fiber orientation, and material properties are critical drivers of ultimate strength and failure modes.

The inclusion of engineered wood products has accelerated progress in this field. Plywood, in particular, has become ubiquitous in construction due to international standards that regulate its manufacture and ensure specified physical and chemical properties [38,39]. Furthermore, the final performance of the panels is highly dependent on the quality of the veneer manufacturing process itself, where factors like the dynamic stability of the peeling lathe are crucial for ensuring uniform veneer thickness [40].

Recent advances have improved the characterization of plywood through both numerical and experimental approaches. Finite element modeling has shown the ability to capture its anisotropic and dynamic response but requires reliable experimental data for validation [41]. Complementary miniaturized shear tests developed in Tuninetti et al., 2024 [42], have confirmed the orthotropic nature of plywood and generated detailed datasets across in-plane and through-thickness directions, suitable for validating advanced models. Within this synergy, plywood presents significant potential for modular applications. The WikiHouse Skylark system, developed in 2020, represents an innovative case study: an open-access digital manufacturing platform for low-rise residential buildings that employs CNC-machined 18 mm plywood panels [43,44]. The system is rooted in a do-it-yourself ethos, offering editable predesigned models tailored to users’ needs. It reduces the reliance on screws, nails, and specialized fasteners through wood–wood joinery, with modules connected by TIE joints that ensure structural integrity against lateral loads.

Granello et al. [43] evaluated the Skylark–WikiHouse system using spruce plywood, performing full-scale beam tests and developing an analytical model. They reported ultimate loads of 21.3–24.9 kN with ductile failure governed by joints. Their study emphasized the need for further research on material plastic behavior and fire performance, as well as comprehensive structural models that account for beams, columns, shear walls, and foundations under service and dynamic loads. Such models require precise experimental data on material properties and boundary conditions. In Chile, the dominant plywood source is Pinus radiata, a softwood with lower mechanical properties than hardwoods such as birch or beech (additional citations needed here). This creates a challenge, as its orthotropic mechanical properties remain insufficiently documented. The proper characterization of strength and stiffness is essential for engineered wood products, including both Laminated Veneer Lumber (LVL) [45] and plywood [42]. Current manufacturing standards classify plywood mainly by bracing function (parallel and perpendicular forces relative to face veneer grain), rather than full orthotropic characterization [38,46,47]. This knowledge gap increases uncertainty and limits reliable numerical modeling.

Fracture prediction in wood-based composites remains a complex challenge, despite the extensive research that exists for other composite materials [48,49]. Advanced fracture prediction strategies often involve micromechanical modeling to predict fracture at small scales [50]. In wood-based materials, this complexity is amplified by their anisotropic nature and by manufacturing variables such as the veneer peeling process [40]. Interfacial bonding quality between veneers is also critical and can be accurately assessed through specialized techniques like acoustic emission [51]. Furthermore, achieving accurate numerical predictions requires overcoming computational challenges like mesh-dependent results in fracture simulations [52], where advanced inverse characterization strategies have proven effective in calibrating robust constitutive models for highly anisotropic materials [53]. Although modeling and calibration techniques have advanced, numerical models for modular engineering designs must be informed and validated by adequate experimental data to ensure their accuracy and extend their applicability.

While foundational work by Granello et al. [43] provided the first structural evaluation of the WikiHouse Skylark system using European spruce plywood, a significant knowledge gap remains for other globally important wood species and performance-enhanced panels. The present study extends this knowledge base in three critical ways: (1) it provides the first experimental data for TIE joints fabricated from Pinus radiata, a softwood widely used in South America and other regions; (2) it quantifies the trade-offs in strength and ductility introduced by commercially available weather-resistant (HR) and fire-retardant (FR) treatments; and (3) it generates a region-specific dataset essential for the safe adoption and numerical modeling of the WikiHouse system in new geographic contexts, particularly those with demanding seismic codes.

The present study therefore undertakes the first experimental investigation of the static mechanical behavior of WikiHouse Skylark TIE joints fabricated from Chilean Pinus radiata plywood. The research compares a standard structural panel (STD) made with bio-adhesive to two performance-enhanced variants: a weather-resistant (HR) panel and a fire-resistant (FR) panel. The specific objectives are as follows: (1) to determine the load–displacement response and ultimate capacity of the TIE joint under monotonic tension and shear loading; (2) to identify and characterize the failure modes associated with each material type and loading condition; and (3) to generate a high-fidelity dataset to fill a critical knowledge gap and provide essential validation for future numerical modeling of the WikiHouse system.

## 2. Materials and Methods

### 2.1. Plywood Panels

The plywood employed in this study was supplied by EAGON Lautaro S.A., located in the Araucanía Region of Chile, Lautaro, Chile. All panels were manufactured using a bio-adhesive, in accordance with the company’s standard production practices. Three types of 18 mm thick, 7-ply Pinus radiata plywood were selected, as summarized in Table 1. Each panel consisted of seven veneers, with four oriented parallel to the panel’s major length (face, back, and two cores) and three oriented perpendicularly, thereby providing a balanced cross-laminated structure.

The first material, hereafter Standard (STD), was a structural-grade panel with a C-grade face veneer and a D-grade back veneer [54]. Veneer grades follow an A–D classification, where Grade A denotes the highest surface quality and Grade D the lowest, determined by the frequency and severity of defects and repairs. The second material, the Weather-Resistant (HR) panel, received a hydro-repellent treatment applied to its faces and edges and complied with ASTM G154 [55] and ASTM D5590 [56], standards that evaluate accelerated weathering and fungal resistance, respectively. The HR panel was also structural-grade, with C/D veneer grading [57]. The third material, the Fire-Resistant (FR) panel, underwent a pressure impregnation fire-retardant treatment that ensured full-volume penetration. The FR plywood achieved a Class B rating under ASTM E84 [58], with a Flame Spread Index (FSI) of 50 and a Smoke Developed Index (SDI) of 75. Unlike the other two materials, the FR panel had higher-quality B/C veneer grading [59], introducing an additional variable that could influence mechanical performance independent of chemical treatment. It is important to note that veneer quality is classified progressively from A (highest quality) to D (lowest quality), based on the number and size of defects such as fissures, cracks, knots, and repairs [39]. Although such “defects” are inherent to the organic nature of wood and the manufacturing process of plywood, they do not necessarily represent the overall behavior of the material. However, they must be considered when interpreting results, as they influence stress distribution [41]. Consequently, only specimens with minimal or no visible defects on both faces were selected for testing.

The differences among veneer grades, treatments, and compliance with standards are detailed in Table 1. These variations were expected to influence the load-bearing capacity, ductility, and failure modes of the joints, and were therefore considered when interpreting results.

### 2.2. Specimen Design and Fabrication

The geometry of the TIE joint assembly was designed at full scale, consistent with the WikiHouse Skylark modular system. Each assembly comprised three CNC-routed components: two identical outer “lock” elements and one central “TIE” element. The joint configuration was intended to reproduce in-service conditions where TIE connections transfer loads between adjacent structural modules.

To evaluate potential anisotropy effects, the central TIE element was fabricated with two veneer orientations relative to the applied load: parallel (//) and perpendicular (⊥). This consideration allowed for a direct assessment of the plywood’s in-plane orthotropy on joint performance. The specimen dimensions were governed by the working space of the universal testing machine and by the need to maintain sufficient clearance around the TIE element to enable visual observation of crack initiation and propagation during testing. To mitigate boundary effects, the edge distance was set to exceed the width of the central element. Only specimens with minimal visible surface defects were selected to avoid bias introduced by outlier flaws.

**Figure 1:**
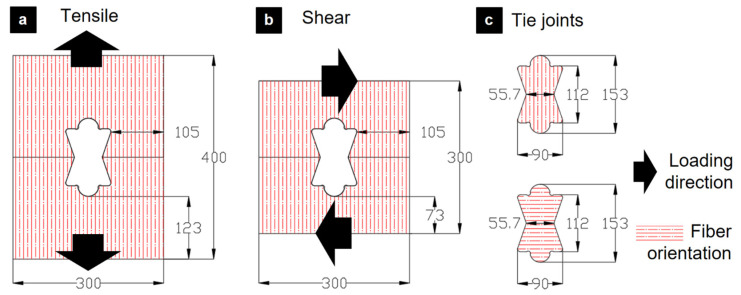
Geometry of the TIE joint assemblies. (**a**) Tensile configuration. (**b**) Shear configuration. (**c**) Central TIE element showing veneer orientation parallel (//) and perpendicular (⊥) to load used for mechanical testing.

The two loading modes investigated were monotonic tension and monotonic shear, both of which represent fundamental stresses experienced by modular connections under service conditions. The complete geometrical configuration is presented in Figure 1, which also illustrates the orientation variations in the TIE element.

The experimental program was designed to systematically evaluate the influence of plywood treatment and veneer grain orientation. For each plywood type (STD, HR, FR), a total of eight specimens were tested: four under monotonic tension and four under monotonic shear. Within each loading condition, two specimens had the TIE element oriented with its face veneer parallel (//) to the load and two were oriented perpendicular (⊥). This experimental design resulted in a total of 24 tests. As the results presented in Section 3 indicate no significant effect from veneer orientation on ultimate strength, the data for each loading condition were subsequently pooled. This provides a statistically robust sample size of n = 4 for each primary comparison (e.g., STD plywood in tension), allowing for a reliable assessment of material performance.

### 2.3. Testing Setup and Protocol

Custom grips were designed and fabricated to facilitate secure load transfer between the plywood assemblies and the universal testing machine. The grips were constructed from ASTM A36 structural steel [60], selected for its high availability and adequate yield strength. To ensure rigidity while minimizing self-deformation, the sections connecting to the plywood specimens were 6 mm thick, whereas the machine–jaw connection sections were 10 mm thick. This arrangement enabled stable attachment to the INSTRON 8801 servohydraulic testing machine, which has a maximum load capacity of 100 kN.

All tests were performed in a controlled laboratory environment at 20 ± 2 °C, with specimens conditioned to a moisture content of 10.5 ± 0.5%. These conditions ensured that moisture variability, a critical parameter for wood-based materials, did not confound the mechanical results. This standard approach was chosen to establish a repeatable experimental baseline, allowing for the direct and unambiguous comparison of the different plywood treatments by isolating their effects from environmental variables.

The experiments followed a displacement-controlled monotonic loading protocol. The applied load was continuously measured by the machine 100 kN load cell. Given the complex geometry of the joint, a standard clip-on extensometer was not applicable. Instead, displacement was measured directly from the crosshead movement. The strain rate was defined relative to the initial length (l_0_) of the narrowest section of the TIE element, and limited to l_0_ × 10^−3^ mm/s, corresponding to a constant crosshead velocity of 0.055 mm/s. This quasi-static rate ensured mechanical equilibrium throughout the test [61,62].

**Figure 2:**
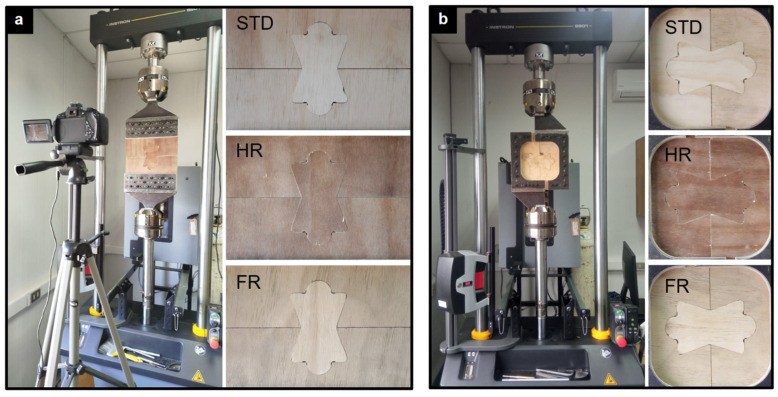
Experimental setup and representative specimens. (**a**) Tension test and (**b**) shear test configurations.

Each specimen was loaded until a maximum displacement of 14 mm was reached. This value was chosen to ensure complete failure and to capture the post-peak nonlinear response of the joint. Recording this softening behavior was deemed essential to distinguish ductile from brittle responses and to assess the influence of panel treatments on load redistribution. The experimental setup for tension and shear is presented in Figure 2.

## 3. Results

### 3.1. Monotonic Tensile Response

**Figure 3:**
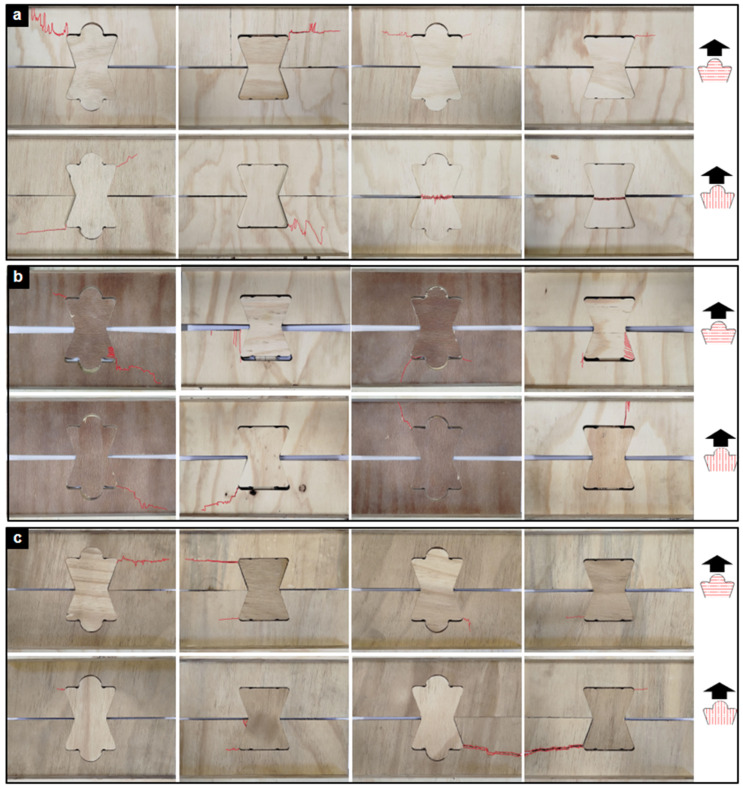
Failure modes of TIE joints under monotonic tensile loading. (**a**) STD plywood. (**b**) HR plywood. (**c**) FR plywood. Each row shows representative fractures under perpendicular (⊥) and parallel (//) orientations. Red lines indicate primary fracture paths.

Representative tensile failure modes are shown in Figure 3. For STD joints (Figure 3a), fractures typically initiated at the inner corners of the outer lock elements, where stress concentrations were highest. In some cases, failure occurred entirely within the central TIE element, producing a classic tensile rupture across the narrowest cross-section.

The HR joints (Figure 3b) also showed corner-initiated fractures, but their response was more complex, often combining tensile cracking with localized shear-crushing at the contact surfaces between TIE and lock elements. Crushed wood fibers and local rearrangement were clearly visible, indicating compound failure mechanisms.

By contrast, FR joints exhibited more stable crack propagation (Figure 3c), oriented normal to the applied tensile load. This pattern suggests that the fire-retardant treatment modified the fracture process, producing a distinctly brittle response with less capacity for redistribution.

**Figure 4:**
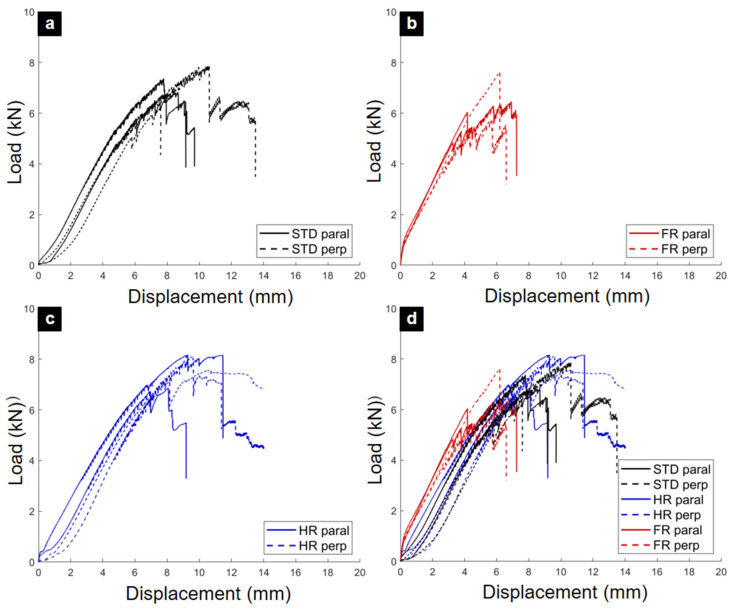
Force–displacement curves from monotonic tensile tests. (**a**) STD plywood. (**b**) HR plywood. (**c**) FR plywood. (**d**) Combined curves for all plywood types.

The load–displacement curves from the tensile tests are shown in Figure 4. All specimens displayed a short nonlinear seating phase of approximately 2–3 mm, attributed to closure of manufacturing tolerances, followed by a quasi-linear elastic response. A characteristic “zigzag” noise appeared near peak load in all cases, likely due to micro-slips and local readjustments within the joint geometry.

The post-peak behavior distinguished the plywood types. STD and HR joints exhibited ductile responses with stepped load drops and gradual capacity decay, indicating redistribution of stresses even after peak failure. In contrast, FR joints lost their load-bearing capacity abruptly, confirming a brittle fracture mode. The aggregated curves (Figure 4d) highlight this contrast in ductility.

A key finding from the tensile tests was that the orientation of the TIE element (parallel vs. perpendicular) did not significantly influence its ultimate strength. This confirms the quasi-isotropic in-plane response of these plywood panels under tensile loading. Based on this result, the data from both orientations were pooled for the final statistical analysis, which is presented in the summary (Table 2).

In terms of strength, HR joints performed best with a mean maximum force (Fmax) of 7674 N, followed by STD joints (7076 N), while FR joints were the weakest (6470 N). The displacement at Fmax further underscores this trend: FR joints reached peak load at only 5.7 mm, compared to 8.6 mm for STD and 9.9 mm for HR, quantitatively confirming their brittle behavior.

### 3.2. Monotonic Shear Response

**Figure 5:**
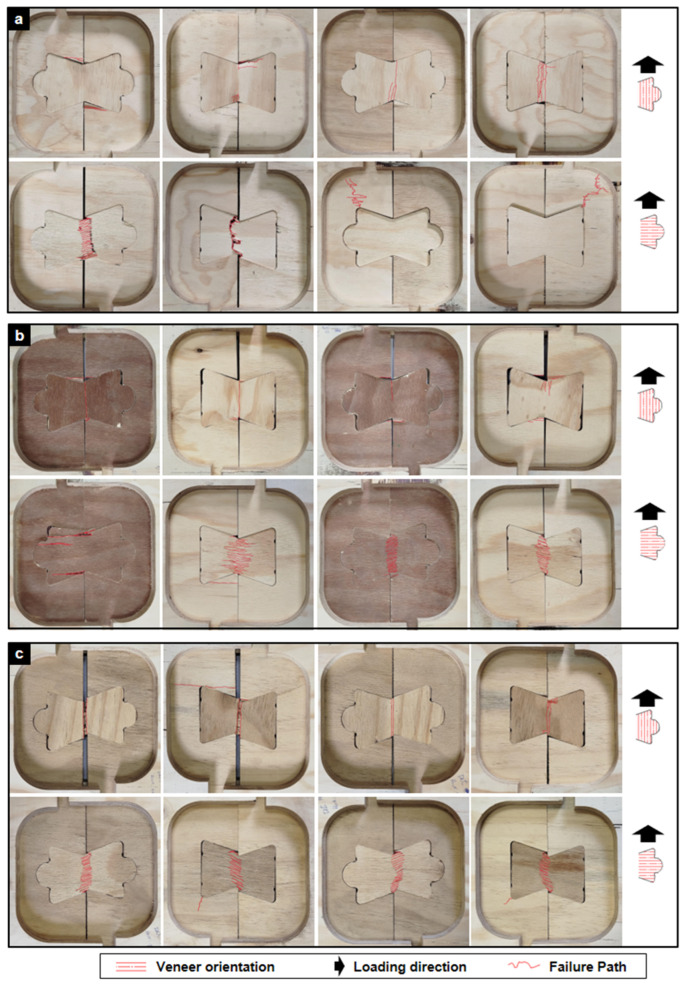
Failure modes of TIE joints under monotonic shear loading. (**a**) STD plywood. (**b**) HR plywood. (**c**) FR plywood. Each row shows representative fractures under perpendicular (⊥) and parallel (//) orientations.

Representative shear failure patterns are shown in Figure 5. The STD joints displayed the greatest variability, including plywood crushing at contact zones, diagonal tension cracking, and shear rupture through the central TIE element, often influenced by fiber orientation.

The HR joints were more consistent, with failures dominated by splitting of the central TIE element and occasional veneer delamination. Minor crushing at the lock element corners was also observed.

The FR joints showed the most uniform behavior: nearly all specimens failed by a clean, brittle fracture across the central TIE section. This homogeneity indicates a predictable, though less ductile, structural response.

**Figure 6:**
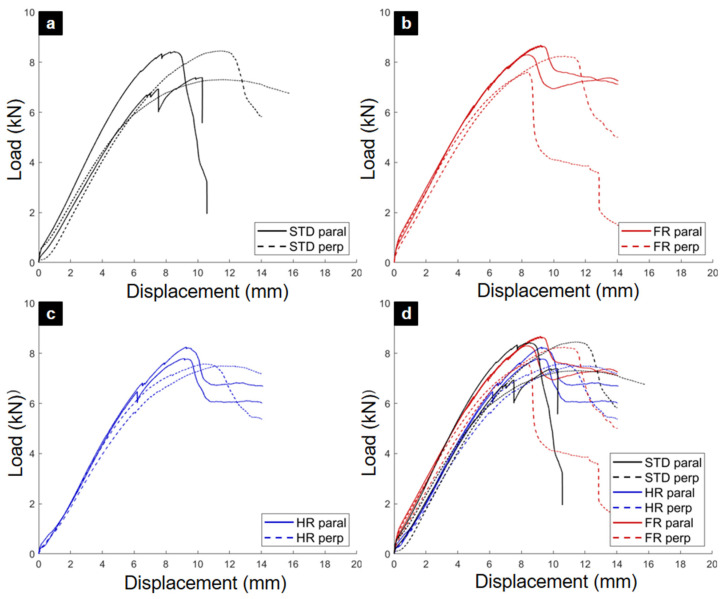
Force–displacement curves from monotonic shear tests. (**a**) STD plywood. (**b**) HR plywood. (**c**) FR plywood. (**d**) Combined curves for all plywood types.

The shear load–displacement curves are presented in Figure 6. The STD specimens exhibited large variability in stiffness and peak load, consistent with their heterogeneous failure patterns. HR specimens showed intermediate variability, with some parallel-oriented TIE elements sustaining gradual post-peak load decay. By contrast, FR specimens displayed highly consistent behavior in their elastic region, in line with their uniform brittle fracture.

Figure 6d, which aggregates all results, demonstrates that while initial stiffnesses were broadly similar, the peak loads and post-peak responses diverged significantly among the plywood types.

The quantitative results from shear testing are provided in Table 2. The results from parallel and perpendicular tests were pooled (n = 4) to computed mean maximum load (Fmax), standard deviation (SD), and coefficient of variation (CV) for each primary test condition. The coefficient of variation, which measures relative variability, was found to be in the range of 4.3% to 12.7%, indicating good reproducibility for this type of component testing. Unlike the tensile results, the FR joints achieved the highest mean Fmax (8207 N), followed by STD (7897 N) and HR (7779 N). This inversion underscores the distinct effects of the fire-retardant treatment under different loading modes.

Nevertheless, FR joints again proved the least ductile, reaching their peak load at an average displacement of only 9.2 mm, compared with 10.5 mm for STD and 10.1 mm for HR. This confirms a persistent strength–ductility trade-off associated with the FR treatment, regardless of loading configuration.

## 4. Discussion

The experimental program provides insight into how material properties influence the mechanical response of the Skylark TIE joint. A key benchmark for interpreting the results is the study by Granello, who reported tensile capacities between 7.4 and 9.0 kN [63] and shear strengths between 11.1 and 12.6 kN [64]. In comparison, the present work using Pinus radiata plywood yielded lower ranges of 6.2–8.2 kN in tension and 7.3–8.4 kN in shear for the STD and HR panels. While this difference can be partly attributed to the species-dependent properties of the wood, the shear performance is also governed by the adhesive system bonding the veneers. The strength of the adhesive bondline is known to be a complex variable sensitive to external factors, which can ultimately influence the composite’s failure mechanism [65]. Whereas Granello [63,64] employed material from Metsä Wood, likely birch or spruce [66], Pinus radiata is a softwood with lower density and stiffness, which directly affects both strength and ductility. These findings underscore the importance of establishing regionally specific datasets for engineered wood products, rather than relying on generic values.

The results also demonstrate the sensitivity of joint behavior to chemical treatments. The hydro-repellent (HR) modification produced negligible differences compared to STD panels, indicating that surface-level treatments may improve durability without compromising structural performance. By contrast, the fire-retardant (FR) treatment induced a marked shift in fracture behavior: specimens exhibited higher shear resistance but a distinctly brittle tensile response. The brittle fracture patterns observed in the FR joints suggest that the treatment affected the material’s failure mechanisms, reducing its ability to dissipate energy. This loss of ductility is of particular concern, as it changes the failure mode from a progressive, warning-based mechanism to a more abrupt and sudden fracture.

The distinct failure modes can be linked to the microstructural effects of the treatments. The hydro-repellent (HR) treatment is typically a surface-level application of a wax emulsion, which has a negligible effect on the internal wood fiber and lignin structure. This explains why its mechanical behavior and ductile failure mode remained similar to the untreated STD panels. In contrast, the fire-retardant (FR) treatment involves pressure impregnation of chemical salts (e.g., phosphates, borates) into the wood’s cellular structure. These salts crystallize within the cell lumens and can stiffen the cell walls, but also lead to embrittlement of the cellulose fibers and potential degradation of the lignin matrix that binds them. This induced material brittleness at the micro-scale is the likely cause of the lower ductility and abrupt fracture patterns observed at the component level.

The new statistical analysis, summarized in Table 2, provides a more robust basis for interpreting the results. The coefficient of variation (CV) for the ultimate load ranged from 4.3% to 12.7%, indicating a level of variability common for tests on engineered wood products. To test for significant differences among the three panel types, a one-way Analysis of Variance (ANOVA) was conducted for each loading condition. The analysis showed no statistically significant difference in ultimate strength for either tensile loading (F(2, 9) = 2.78, *p* = 0.115) or shear loading (F(2, 9) = 0.82, *p* = 0.47). This indicates that while the mean strength values varied, the differences were not large enough to be considered a true effect of the treatments, likely due to the inherent material variability and the sample size of the study.

The pronounced loss of ductility in the FR panels warrants further discussion. The pressure impregnation process may create a gradient of chemical concentration and material properties through the panel’s thickness, leading to mechanical inhomogeneity. This phenomenon is analogous to findings in other material systems, where variations in properties across a cross-section can dictate fracture behavior. For instance, He et al. [67] demonstrated that inhomogeneities in ductile iron pipes significantly influence their mechanical response and failure initiation. Similarly, if the fire-retardant treatment renders the outer veneers more brittle than the core, it could promote premature crack initiation at the surface under tensile loading, leading to the observed abrupt, low-ductility failure. This highlights that the uniformity of treatment is as important as its chemical function.

Another important outcome is the observation that the orientation of the central TIE element (parallel vs. perpendicular to the load axis) did not significantly affect ultimate strength. This quasi-isotropic in-plane behavior validates the assumption often made for plywood-based structural elements and simplifies the modeling approach for modular systems. It indicates that variability in connection performance is more strongly driven by treatment effects and local stress concentrations than by fiber orientation.

To contextualize the performance from a structural design perspective, the measured capacities can be compared to established values for timber connectors. For instance, design guides for dowel-type connections in plywood often list characteristic shear capacities in the range of 2–5 kN per fastener. Our TIE joints, exhibiting mean shear capacities between 7.8 kN and 8.2 kN, demonstrate a strength that is comparable to or exceeds that of multiple conventional fasteners. This preliminary comparison suggests that the WikiHouse TIE joint is a structurally viable connection, although a full design-value derivation according to standards like Eurocode 5 would require additional testing and analysis [68].

It is important to note that the results and conclusions of this study are specific to the controlled laboratory environment in which the tests were conducted. As moisture content significantly influences the mechanical properties of wood, the performance of these TIE joints under high-humidity, in-service conditions may differ. Therefore, the data presented here should be considered an essential baseline characterization, providing the foundational data required for future, more complex investigations into environmental effects.

The study of new modular construction systems is inherently complex. Similarly, analyzing the structural performance of a building under cyclic or dynamic loads is a demanding task that requires substantial resources [69,70,71]. A widely adopted practical strategy is the local study of mechanical joints within a construction system [72,73,74,75,76,77].

From a methodological standpoint, this approach requires fewer economic and computational resources, smaller physical testing spaces, and provides a detailed understanding of the behavior of the studied joint. Testing joints at the component level proved effective in capturing realistic stress states while reducing resource demands. This approach provided clear evidence of the influence of treatments and failure mechanisms without requiring full-scale structural prototypes. Such data are essential for refining finite element models [78], where accurate characterization of localized connections governs the reliability of larger-scale predictions.

## 5. Conclusions

This experimental investigation of the WikiHouse Skylark TIE joint fabricated from standard and performance-enhanced Pinus radiata plywood establishes an essential baseline for its static mechanical behavior. The main conclusions are as follows:

- Performance-enhancing treatments significantly influence joint behavior, particularly the failure mode and ductility. Their selection must balance durability and fire resistance against structural impact.
- The hydro-repellent (HR) treatment did not negatively impact the joint’s mechanical performance. Both STD and HR joints exhibited a desirable ductile failure mode with gradual load decay, though no statistically significant difference in their ultimate strengths was found.
- The fire-retardant (FR) treatment introduced a critical strength–ductility trade-off. While the mean shear strength was the highest observed (8207 N), the treatment induced a brittle fracture mode and a significant loss of ductility. This change from a ductile to brittle failure raises important considerations for structural design, especially in seismic regions.

Overall, the lower strengths obtained for Pinus radiata compared to values reported for birch or spruce highlight the need for species-specific and treatment-specific characterization, since reliance on the generic literature data may lead to unreliable design assumptions. The dataset and failure analyses reported here provide a robust empirical basis for developing high-fidelity finite element models, which are essential for accurate structural simulations of modular timber systems. In turn, these models can support the safe and code-compliant implementation of open-source construction platforms such as WikiHouse in seismic regions.

While this study has established a critical baseline under static monotonic loading, future research must extend this characterization to more complex conditions. A crucial next step is to investigate the performance of the TIE joints under a wide range of in-service moisture contents to quantify the effect of humidity. Furthermore, cyclic and dynamic tests are required to evaluate the seismic and wind performance, thereby broadening the structural applicability and ensuring the safe implementation of this modular timber solution.

## Figures and Tables

**Table 1:** Main specifications and properties of the three 18 mm, 7-ply Pinus radiata plywood panels tested: Standard (STD), Weather-Resistant (HR), and Fire-Resistant (FR).

Property	STD	HR	FR	Method/Reference
Nominal Thickness (mm)	18	18	18	Manufacturer Specification
Number of Plies	7	7	7	
Veneer Grade (Face/Back)	C/D	C/D	B/C	
Special Treatment	None	Hydro-repellent	Fire-retardant	
Standards Met	PS 1–22	ASTM G154, ASTM D5590	ASTM E84 (Class B)	
Mean Density (kg/m^3^)	520	525	580	
Moisture Content (%)	10.5 ± 0.5	10.5 ± 0.5	10.5 ± 0.5	Conditioned Lab Env.
Orthotropic Shear Properties	See Ref. [42]			Tuninetti et al., 2024

**Table 2:** Summary of Monotonic Test Results for TIE Joints (n = 4).

Plywood Type	Loading Mode	Mean Fmax (N)	Std. Dev. (N)	CV (%)
Standard	Tension	7076	724	10.2
	Shear	7897	633	8
Fire-Resistant	Tension	6470	822	12.7
	Shear	8207	445	5.4
Weather-Resistant	Tension	7674	606	7.9
	Shear	7779	338	4.3

## Data Availability

The original contributions presented in this study are included in the article. Further inquiries can be directed to the corresponding authors.
